# A Study on the Design Method of Indoor Fine Particulate Matter (PM2.5) Pollution Control in China

**DOI:** 10.3390/ijerph16234588

**Published:** 2019-11-20

**Authors:** Qingqin Wang, Dongye Fan, Li Zhao, Weiwei Wu

**Affiliations:** China Academy of Building Research, Beijing 100013, China; fandongye@126.com (D.F.); zhaolicabr@163.com (L.Z.); weiweiwucqu@163.com (W.W.)

**Keywords:** fine particulate matter, design method, equipment selection, pollution control measures

## Abstract

Airborne fine particulate matter (PM2.5) is an important factor affecting indoor air quality and is significantly related to human health. A lot of research has been done on PM2.5 pollution control in buildings, but there is lack of systematic indoor PM2.5 pollution control for engineering applications. In this study, first, we propose an outdoor calculated concentration of PM2.5 in 74 cities, in China, using the “not guaranteed for five days” method, which is based on outdoor PM2.5 concentration monitoring data from 2016 to 2018. Then, different levels of indoor calculated concentrations of PM2.5 (25 μg/m^3^, 35 μg/m^3^, and 75 μg/m^3^) are put forward. Secondly, methods for selecting air purification equipment in centralized, semi-centralized, and decentralized air conditioning systems are proposed. Finally, PM2.5 pollution control measures and system energy-saving operation strategies in buildings are put forward. This study stipulates the calculation of PM2.5 concentration, unifies design methods, proposes control measures, and standardizes operations management. The aim of this study is to provide technical guidance for engineering design, construction and operation, and therefore to reduce the risk of indoor diseases caused by exposure to PM2.5.

## 1. Introduction

Human health can significantly be affected by particulate matter. Long-term exposure to an environment that is polluted by particulate matter can damage the human respiratory system, cardiovascular and cerebrovascular system, immune system, nervous system, and reproductive system, etc. [1,2,3,4,5]. EUROVENT 4/23-2018 Selection of EN ISO 16890 Rated Air Filter Classes for General Ventilation Applications pointed out that particles with a diameter of 10 μm or smaller can reach the respiratory duct, potentially causing decreased lung function. Particles with a diameter of 2.5 μm or smaller can penetrate the lungs, causing decreased lung function, skin, and eye problems. Particles with a diameter of 1 μm or smaller are tiny enough to enter the bloodstream, leading to cancer, cardiovascular diseases, and dementia [6]. According to the data from the World Health Organization (WHO), around seven million people die every year from exposure to fine particles in polluted air that penetrate deep into the lungs and cardiovascular system, causing diseases including stroke, heart disease, lung cancer, chronic obstructive pulmonary diseases, and respiratory infections, including pneumonia [7]. The WHO’s Air Pollution and Child Health: Prescribing Clean Air shows that 93% of the world’s children under 15 years of age are exposed to ambient PM2.5 levels above the WHO air quality guidelines, which include 630 million children under five years of age, and 1.8 billion children under 15 years of age [8]. The WHO air quality guidelines estimate that reducing annual average PM2.5 concentrations from levels of 35 μg/m^3^, common in many developing cities, to the WHO guideline level of 10 μg/m^3^, could reduce air pollution-related deaths by approximately 15% [9].

**Figure 1:**
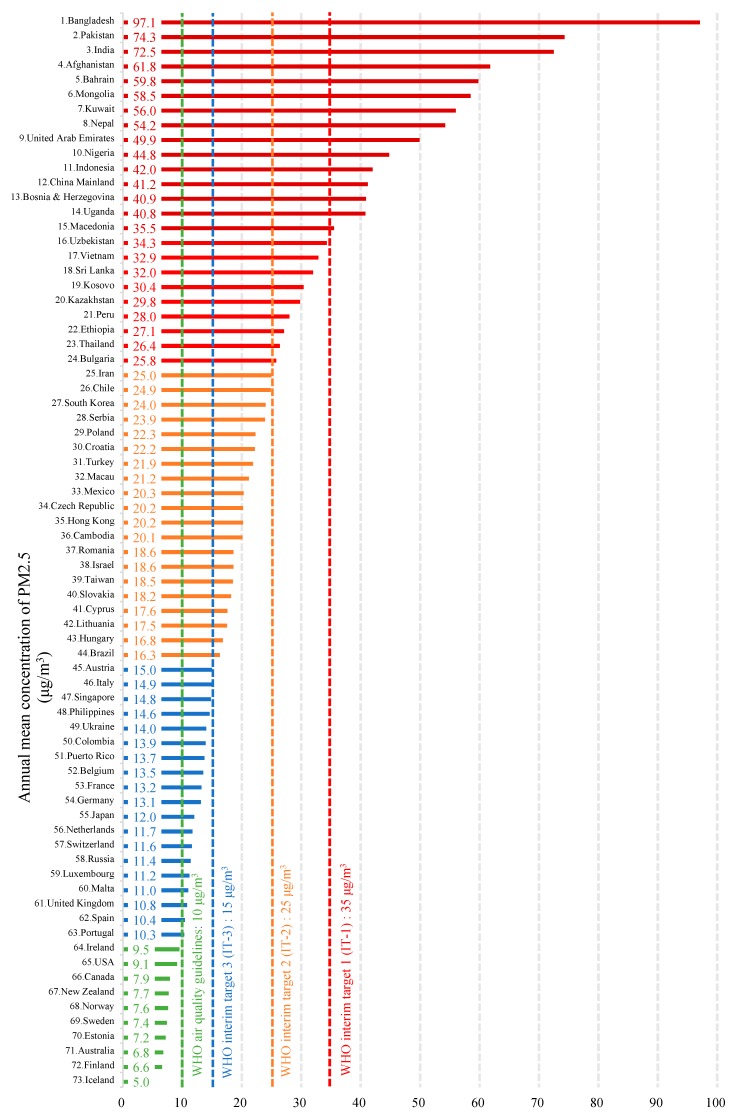
Outdoor particulate matter (PM2.5) pollution in 73 regions, in 2018 [10].

**Figure 2:**
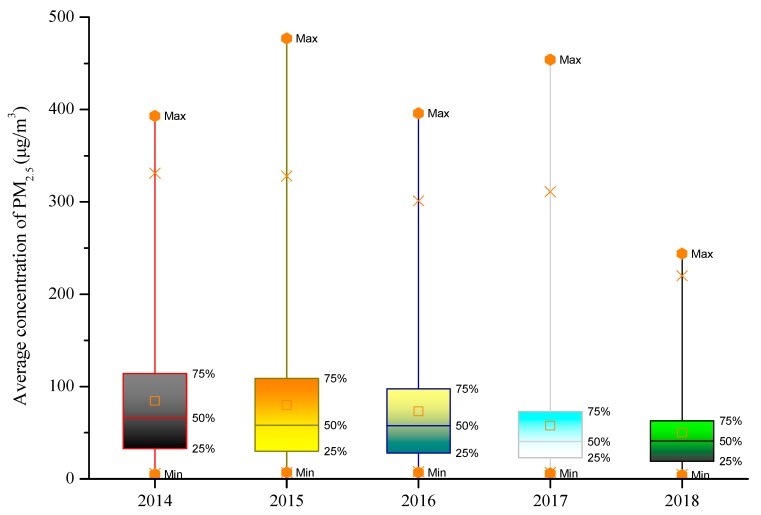
The variation of outdoor PM2.5 average concentration in Beijing, from 2014 to 2018.

**Figure 3:**
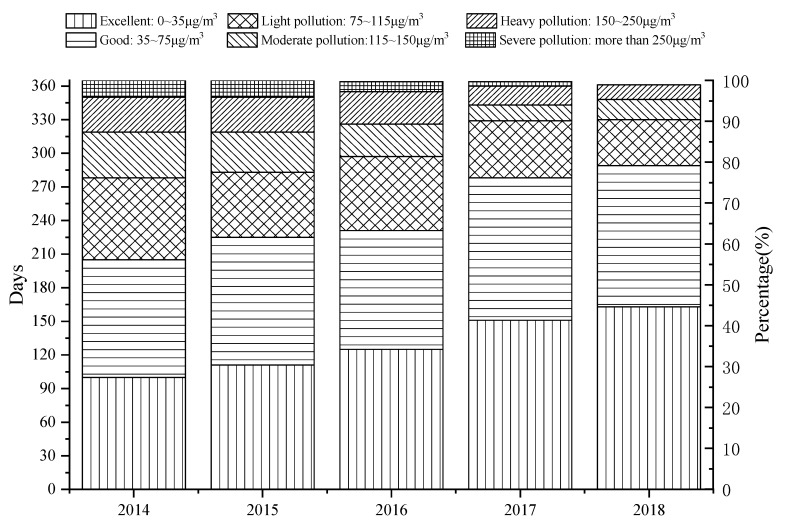
2014~2018 outdoor PM2.5 pollution distribution in Beijing. Note: The data in Figure 2 and Figure 3 are derived from the China Air Quality Online Monitoring and Analysis Platform. There is no monitoring data on April 16, 2016, September 6, 2016, May 4, 2017, March 28, 2018, and May 5, 26, and 28, 2018, and the corresponding data are not counted.

Currently, air pollution is still a challenging issue in many parts of the world. The 2018 World Air Quality Report shows that in only 10 regions out of the 73 in the world, the annual average PM2.5 concentration does not exceed 10 μg/m^3^. The annual average PM2.5 concentration in China’s mainland is 41.2 μg/m^3^, which is ranked 12th among the 73 regions (Figure 1) [10]. In 2013, China began to monitor atmospheric PM2.5 concentrations in key areas such as Beijing-Tianjin-Hebei, Yangtze River Delta, and Pearl River Delta, as well as municipalities and provincial capitals, to evaluate the concentration levels based on the Ambient Air Quality Standard, GB 3095-2012, for PM2.5 concentrations. The GB 3095-2012 standard stipulates that the annual average concentration limit of nature reserves, scenic spots, and other areas that require special protection is 15μg/m^3^, and the daily average concentration limit is 35 μg/m^3^. The annual average concentration limit for residential, commercial residents, cultural, industrial, and rural areas is 35 μg/m^3^, and the daily average concentration limit is 75 μg/m^3^ [11]. China has made positive progress in controlling air pollution over recent years. For instance in Beijing, the annual average concentration of outdoor PM2.5 decreased from 85 μg/m^3^, in 2014, to 50 μg/m^3^, in 2018, i.e., a 41.2% reduction (Figure 2). The number of days for the PM2.5 concentration in a range of 0~35 μg/m^3^ has increased from 100 to 163 days, and for the range of 35~75 μg/m^3^ the number of days has increased from 105 to 126 days (Figure 3). However, compared with developed countries, there is still a significant gap between China’s outdoor air quality and standard limits.

It is still difficult for the outdoor PM2.5 concentration in China to meet the standard levels in the short term. The indoor PM2.5 pollution has the characteristics of easy control, long exposure time, and significant health effects. Effective control of PM2.5 pollution in buildings can significantly reduce the risk of diseases by PM2.5. This paper introduces the following: (1) Outdoor and indoor calculated concentrations of PM2.5, (2) design and calculation methods of PM2.5 pollution control in buildings, (3) a selection method for air purification equipment in different air conditioning systems, (4) PM2.5 pollution control measures in buildings, and (5) an energy-saving operation strategy for the PM2.5 pollution control system. Furthermore, the China Academy of Building Research has formulated the China Engineering Construction Standardization Association standard entitled the T/CECS 586-2019 Technical Specification for Pollution Control of Fine Particulate Matter (PM2.5) in Buildings. This standard is applicable to indoor PM2.5 pollution control in built, rebuilt, and expanded civil buildings in China.

## 2. Calculation Parameter of Indoor PM2.5 Pollution Control

### 2.1. Outdoor Calculated Concentration of PM2.5

Indoor PM2.5 pollution control design is similar to the design of heating and air conditioning systems that provide desirable indoor air quality. The control of PM2.5 does not have a significant impact if it deviates from design requirements in a short period of time. Therefore, selecting the outdoor PM2.5 concentration under a certain guaranteed rate as the outdoor design concentration, can ensure that the indoor PM2.5 concentration will meet the requirements within the required number of days throughout the year. Additionally, it avoids common problems such as “large” or “small” selection of air purification equipment caused by different calculation methods such as annual mean, 24 h mean, and experience values [12,13,14].

The outdoor calculated concentration of PM2.5 should adopt an average daily mass concentration that is not guaranteed for five days in the past three years. That is, the average mass concentration of PM2.5 per year in each statistical year in descending order should be arranged, and then the highest five days average mass concentration of PM2.5 should be removed. The average mass concentration of PM2.5 on the sixth day is the daily mass concentration that is not guaranteed for five days. The average daily mass concentration not guaranteed for five days in the past three years is the annual average mass concentration of PM2.5. The outdoor calculated concentrations of PM2.5, in 74 cities, are shown in Table 1. For cities which are not listed in this table, outdoor calculated concentration of PM2.5 in the nearest city is recommended.

### 2.2. Indoor Calculated Concentration of PM2.5

The indoor calculated concentration of PM2.5 is divided into two categories, current value and guiding value. The current value is the daily average mass concentration of PM2.5, which is referenced from the current national standards in China. The guiding value is the recommended daily average mass concentration of PM2.5, which is recommended by the international standard. The details are listed in Table 2.

The outdoor PM2.5 concentration limit (daily average) specified in the Ambient Air Quality Standard, GB 3095-2012, is the Class I concentration limit which is 35 μg/m^3^, and the Class II concentration limit which is 75 μg/m^3^. Considering the current situation of particulate matter pollution and its health risks in China, the current indoor calculated concentrations of PM2.5 in this standard are consistent with the concentration limit defined in the Ambient Air Quality Standard GB 3095-2012. The Class I concentration limit of guiding value is based on the WHO’s 24 h average (25 μg/m^3^), which is based on the safety value of the human body exposure over a 24 h period and annually. The Class II standard is identical to the level 1 concentration limit in the Ambient Air Quality Standard, GB 3095-2012.

Buildings with strict requirements of indoor PM2.5 concentration, such as nurseries, kindergartens, nursing homes, and other special buildings should be designed with a Class I standard value. Buildings with less strict requirements of indoor PM2.5 concentration, such as ordinary residential buildings, office buildings, store buildings, libraries, waiting rooms (halls), etc. should be designed with at least a Class II standard value.

## 3. Theory and Calculation Method of Indoor PM2.5 Pollution Control

The theoretical calculation method for indoor PM2.5 pollution control design is to adopt the basic mass balance equation:

*D* = *M* (1)

where *D* is the PM2.5 removal capacity of the air purification equipment, i.e., the mass of the PM2.5 removed by the air purification equipment per unit time (μg/h); *M* is the indoor PM2.5 load, i.e., the mass of the obtained PM2.5 per unit time (μg/h).

**Figure 4:**
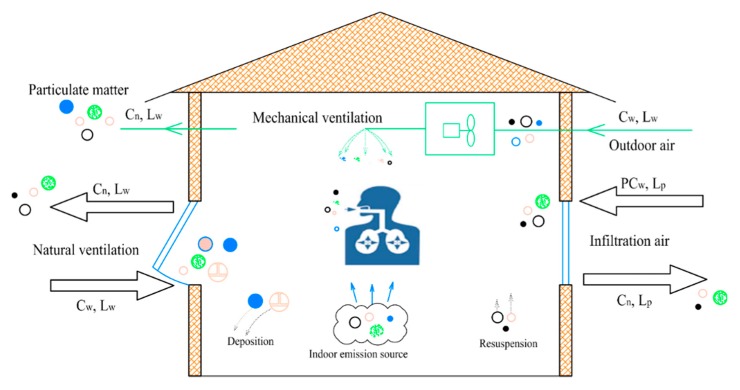
Schematic Map of PM2.5 Sources in Building.

The indoor PM2.5 load is usually generated by three parts, i.e., outdoor air, infiltration air, and indoor emission sources, as demonstrated in Figure 4. In the indoor PM2.5 pollution control design, the doors and windows are generally considered closed, and deposition and resuspension of particulate matters are neglected. The specific calculation is as follows:

- Indoor PM2.5 load is calculated by the equation below:
  (2) $$M=M_{w}+M_{p}+M_{n}$$
  where *M* is the indoor PM2.5 load (μg/h), *M_w_* is the outdoor air PM2.5 load (μg/h), $M_{p}$ is the infiltration PM2.5 load (μg/h), and $M_{n}$ is the indoor sources PM2.5 load (μg/h).
- Outdoor air PM2.5 load is calculated as follows:
  (3) $$M_{w}=\left(C_{w}-C_{n}\right)L_{w}$$
  where $C_{w}$ is the outdoor calculated concentration of PM2.5 (μg/m^3^), $C_{n}$ is the indoor calculated concentration of PM2.5 (μg/m^3^), and $L_{w}$ is the outdoor air volume (m^3^/h).
- The infiltration PM2.5 load is calculated according to the following formula:
  (4) $$M_{p}=\left(PC_{w}-C_{n}\right)L_{p}$$
  (5) $$L_{p}=nV$$
  where *P* is the penetration coefficient, $L_{p}$ is the amount of air that permeates into the room from the gap between the external door and window (m^3^/h), *n* is the rate of air permeation (h^−1^), and *V* is the volume of the room (m^3^).

This research shows that the external window penetration coefficient of PM2.5 is affected by the gap condition, indoor and outdoor pressure difference, and outdoor relative humidity, etc. The influencing factors comprise gap characteristics, i.e., type, size and roughness, overall airtightness of the external window, indoor and outdoor temperature, outdoor wind speed, and relative humidity, etc. Moreover, the penetration coefficients obtained by different researchers are not the same, and the range is distributed mostly between 0.5 and 0.9 [14,15], and therefore the PM2.5 external window penetration coefficient is not a fixed value. We measured the penetration coefficient of PM2.5 external window under different airtight and various pressure conditions. The results are shown in Table 3, which can be used as a reference in design calculations.

The airtightness level of the external window is consistent with GB/T 7106-2008 Graduations and Test Methods of Air Permeability, Watertightness, Wind Load Resistance Performance for Building External Windows and Doors [16].The airtightness level of the external window is graded by two indexes of Q_1_ and Q_2_, as listed in Table 4. Q_1_ refers to the volume of air flow through the unit joint length of the opening part, and Q_2_ refers to the volume of air flow through a unit area. The test conditions are 293 K (20 °C), 101.3 kPa (760 mm Hg), 1.202 kg/m^3^ air density, and the air absolute pressure difference between the indoor and outdoor window is 10 Pa.

The amount of infiltration air into the room from the gap between the external door and window is affected by the outdoor wind speed, indoor and outdoor temperature, as well as orientation. The infiltration air volume varies in different regions and seasons. The commonly used methods for obtaining the infiltration air volume are aperture, air change rate, and test statistics methods. This paper estimates the amount of infiltration air through the external window according to the number of air changes. According to the results of relevant research, when the outdoor wind speed is in the ranges of 0~0.2 m/s, 1.6~3.3 m/s, and 5.5~7.9 m/s, the infiltration change rates of the external window are 0.1 h^−1^, 0.22 h^−1^, and 0.39 h^−1^, respectively [17,18]. It is assumed when the outdoor PM2.5 is severely polluted, the outdoor environment is not windy or breezy. Under this condition, different external airtight windows are measured for the certain ranges of air change rate, as listed in Table 5. The airtightness levels of the external window are in-situ measurements.

• Indoor sources for PM2.5 load

Indoor sources for PM2.5 mainly include equipment operation, personnel movement, smoking, burning, cooking, housework activities, etc. Currently, no globally accepted results have been established for the emission rate of indoor PM2.5 pollution sources. Several tests are carried out on the emission rate of the indoor PM2.5 pollution sources, but the results are subject to ventilation conditions, room size, outdoor PM2.5 concentration, operation form, and some other involved conditions. Consequently, the results are based on the test conditions. Thus, a comprehensive determination of the indoor sources PM2.5 load is required based on the real conditions including the intensity, frequency, simultaneous probability, and removal time of the indoor PM2.5 sources rather than simply recording the data.

It is also recommended to consider the corresponding measures to control the pollution of indoor sources, which are discussed in the Section on Control Measures in this paper. Therefore, the indoor sources PM2.5 load can either be ignored or used directly in design calculations if there is a known indoor sources PM2.5 load.

## 4. Selection of Air Purification Equipment

The ventilation and air conditioning system in a building can effectively control the indoor temperature or humidity to ensure the demand for outdoor air. The PM2.5 pollution control system is a type of air purification system which operates as an important supplement in the operation of the ventilation and air conditioning system. It can effectively control the indoor PM2.5 concentration and protect human health.

The indoor PM2.5 pollution control system, in particular, the modification part in the existing building, should be adapted to the ventilation and air conditioning system to satisfy the relevant requirements of the ventilation and air conditioning system and effectively control the indoor PM2.5 pollution. The ventilation and air conditioning system is divided into the centralized, semi-centralized, and decentralized air conditioning systems based on setting conditions of the air handling equipment. The air purification equipment can flexibly be set for different systems.

### 4.1. Centralized Air Conditioning System

#### 4.1.1. Outdoor Air without Pretreatment

When the outdoor air of the centralized air conditioning system is not pretreated, multiple rooms share the air purification equipment. In this case, the air purification equipment purifies the PM2.5 load of all rooms. The PM2.5 removal capacity of the air purification equipment should not be lower than the total PM2.5 loads of each room.

**Figure 5:**
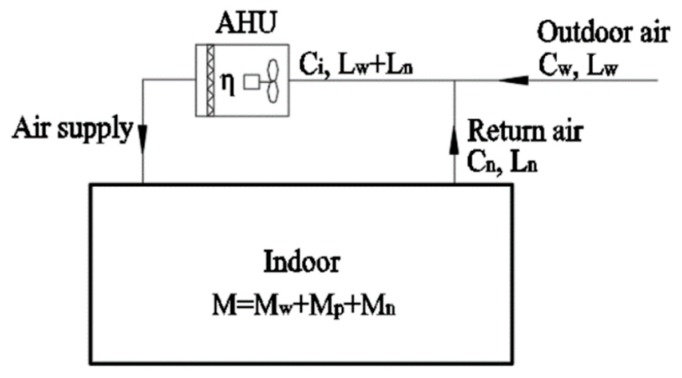
Schematic diagram of a centralized system without outdoor air pretreatment.

Figure 5 shows the principle of a centralized system without outdoor air pretreatment. The formula calculation is carried out as follows:

(6) $$D=C_{i}\cdot (L_{w}+L_{n})\cdot \eta \geq M$$

(7) $$C_{i}(L_{w}+L_{n})=C_{w}\cdot L_{w}+C_{n}\cdot L_{n}$$

where $C_{i}$ is the inlet air PM2.5 concentration (μg/m^3^) of the air purification equipment, η is PM2.5 comprehensive filtration efficiency of the air purification equipment, $L_{w}$ is the outdoor air volume (m^3^/h), and $L_{n}$ is the return air volume (m^3^/h).

The following expression is calculated using Equations (6) and (7):

(8) $$\eta \geq \frac{M}{C_{w}\cdot L_{w}+C_{n}\cdot L_{n}}$$

Selection of the air filter can be done by considering Equation (8). For an air purification equipment with a series of air filters, the PM2.5 integrated filtration efficiency can be calculated as follows:

(9) $$\eta =1-\left(1-\eta_{1}\right)\left(1-\eta_{2}\right)\ldots \left(1-\eta_{m}\right)$$

where $\eta_{m}$ is the PM2.5 filtration efficiency of the *m*th> air filter.

#### 4.1.2. Outdoor Air with Pretreatment

The air purification equipment in the outdoor air handling unit (OAHU) is preferred to purify all of indoor PM2.5 loads when the outdoor air is pretreated.

**Figure 6:**
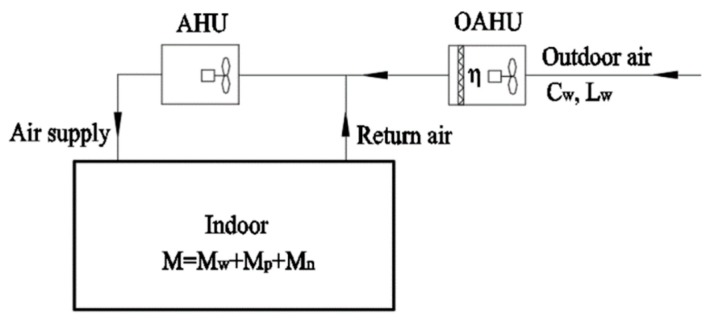
Schematic diagram of a centralized system with outdoor air pretreatment and purifying indoor PM2.5 load.

Figure 6 shows the principle of a centralized system in which the air purification equipment in the OAHU purifies all indoor PM2.5 loads. The formula is as follows:

(10) $$D=C_{w}\cdot L_{w}\cdot \eta \geq M$$

which is:

(11) $$\eta \geq \frac{M}{C_{w}\cdot L_{w}}$$

The selection of air filter in OAHU can be performed according to Equation (11).

If the air purification equipment in the OAHU is unable to purify all of the indoor PM2.5 loads, two schemes can be used for treatment purpose:

(1) The air purification equipment in the OAHU only purifies the outdoor air PM2.5 load, and the air purification equipment in the air handling unit (AHU) purifies the infiltration PM2.5 load and indoor sources PM2.5 load.

**Figure 7:**
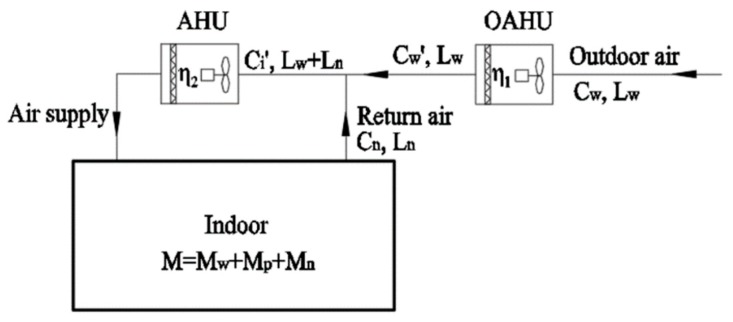
Schematic diagram of a centralized system with outdoor air pretreatment only purifying outdoor air PM2.5 load.

Figure 7 shows the principle of a centralized system in which the air purification equipment in the OAHU only purifies the outdoor air PM2.5 load. The calculation is carried out as follows:

(12) $$D_{w}=C_{w}\cdot L_{w}\cdot \eta_{1}=M_{w}$$

where $\eta_{1}$ is the comprehensive filtration efficiency of the air purification equipment in the OAHU, and $D_{w}$ is the PM2.5 removal capacity (μg/h) of the air purification equipment in the OAHU.

From Equation (12), it can be concluded that:

(13) $$\eta_{1}=\frac{M_{w}}{C_{w}\cdot L_{w}}$$

The air filter selection in the OAHU can be performed according to Equation (13).

(14) $$D_{s}=C_{i}^{\prime }\cdot (L_{w}+L_{n})\cdot \eta_{2}\geq M_{p}+M_{n}$$

(15) $$C_{i}^{\prime }(L_{w}+L_{n})=C_{w}^{\prime }\cdot L_{w}+C_{n}\cdot L_{n}$$

(16) $$\eta_{1}=\frac{C_{w}-C_{w}^{\prime }}{C_{w}}$$

where $\eta_{2}$ is the comprehensive filtration efficiency of the air purification equipment in the AHU, $D_{s}$ is the PM2.5 removal capacity of the air purification equipment in the AHU (μg/h), $C_{i}^{\prime }$ is the PM2.5 inlet air concentration of the AHU (μg/m^3^), and $C_{w}^{\prime }$ is the PM2.5 outlet air concentration of the OAHU (μg/m^3^).

From Equations (14) to (16), the following expression can be concluded:

(17) $$\eta_{2}\geq \frac{M_{p}+M_{n}}{(1-\eta_{1})C_{w}L_{w}+C_{n}L_{n}}$$

The selection of air filter in the AHU can be performed according to Equation (17).

(2) The OAHU only purifies the outdoor air PM2.5 load, partially infiltration PM2.5 load and indoor sources PM2.5 load. Air purification equipment of the AHU purifies the remaining infiltration PM2.5 load and indoor sources PM2.5 load.

In this scheme, the overall filtration efficiency of the air purification equipment in the OAHU is greater than the calculated values by Equation (13) and smaller than the calculated values by Equation (11). In the design selection process, a certain air filter can be selected in the OAHU to obtain its actual comprehensive filtration efficiency.

**Figure 8:**
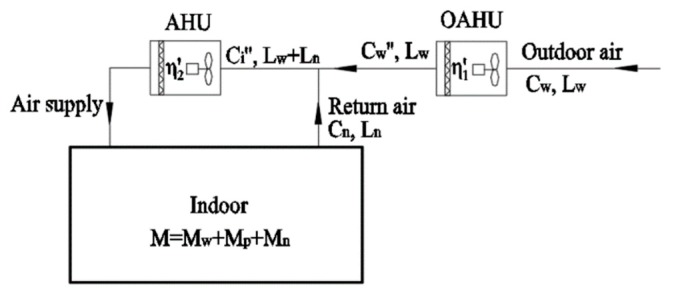
Schematic diagram of a centralized system with outdoor air pretreatment and purifying outdoor air PM2.5 load, partially, infiltrated PM2.5 load and indoor sources PM2.5 load.

The schematic diagram is shown in Figure 8. The required equations are as follows:

(18) $$D_{s}^{\prime }=C_{i}^{\prime \prime }\cdot (L_{w}+L_{n})\cdot \eta_{2}^{\prime }\geq (M-C_{w}\cdot L_{w}\cdot \eta_{1}^{\prime })$$

(19) $$C_{i}^{\prime \prime }(L_{w}+L_{n})=C_{w}^{\prime \prime }\cdot L_{w}+C_{n}\cdot L_{n}$$

(20) $$\eta_{1}^{\prime }=\frac{C_{w}-C_{w}^{\prime \prime }}{C_{w}}$$

From Equations (18) to (20), the following expression can be concluded:

(21) $$\eta_{2}^{\prime }\geq \frac{M-C_{w}\cdot L_{w}\cdot \eta_{1}^{\prime }}{(1-\eta_{1}^{\prime })C_{w}L_{w}+C_{n}L_{n}}$$

Air filter selection in an AHU can be performed according to Equation (21).

### 4.2. Semi-Centralized Air Conditioning System

In a semi-centralized air conditioning system, the air purification equipment in the OAHU should be set up centrally to purify the outdoor air PM2.5 load. Otherwise, the PM2.5 concentration of the outdoor air supply will be higher than the indoor calculated concentration of PM2.5, and the outdoor air will become the source of pollution, which can put more the burden on the air purification equipment. Therefore, the PM2.5 removal capacity of all OAHUs should not be lower than the outdoor air PM2.5 load.

**Figure 9:**
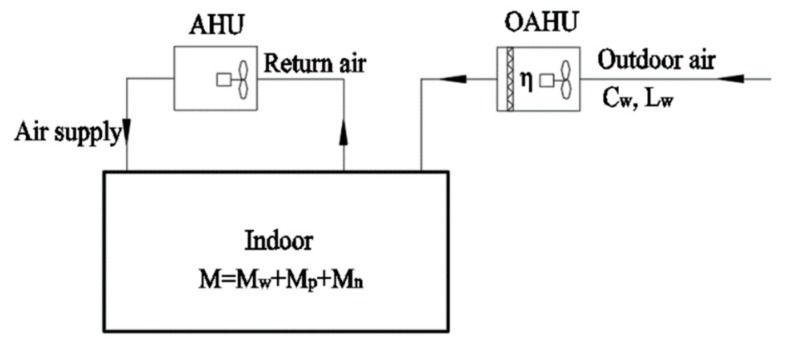
Schematic diagram of a semi-centralized system with outdoor air pretreatment and purifying indoor PM2.5 load.

- Taking all of the indoor PM2.5 loads by the OAHU is preferred (Figure 9). This method can be designed and calculated using Equation (11) and air filter selection.
- When the air purification equipment of the OAHU cannot purify all indoor PM2.5 loads, other air purification equipment should be installed at the end of the ventilation and air conditioning system. The sum of the PM2.5 removal capacity of the AHU and OAHU should not be lower than all indoor PM2.5 loads.

**Figure 10:**
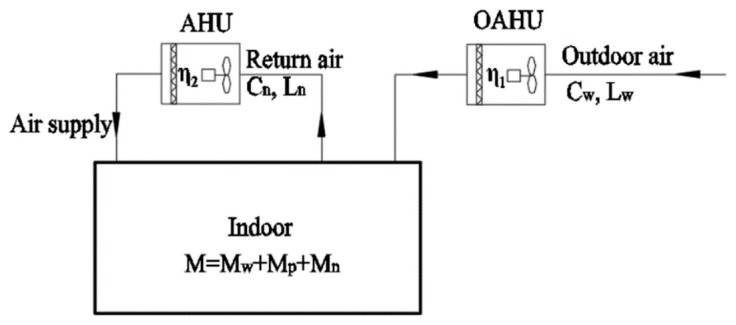
Schematic diagram of a semi-centralized system with outdoor air pretreatment.

The specific design calculation and selection can refer to two schemes of centralized outdoor air pretreatment when the air purification equipment in the OAHU cannot purify all indoor PM2.5 loads. The only difference is that the air intake concentration of the AHU is $C_{n}$ and the air intake volume is $L_{n}$ (see Figure 10).

### 4.3. Decentralized Air Conditioning System

**Figure 11:**
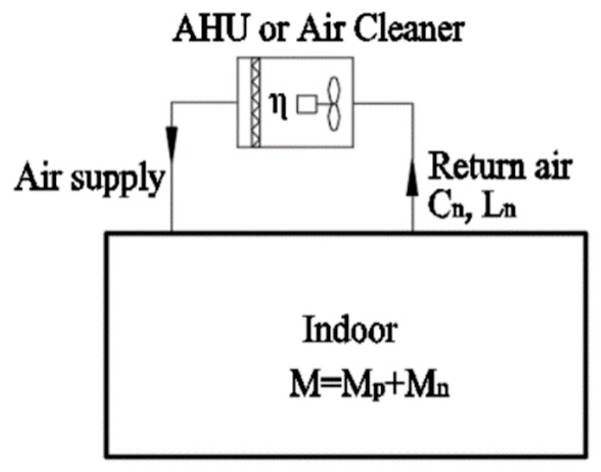
Schematic diagram of the decentralized system.

Since the decentralized system does not concentrate on outdoor air, it usually purifies the indoor PM2.5 load by installing a low-resistance and high-efficiency air filter or a separate air cleaner at the end of the air conditioner. This system is often used in residential buildings. In this case, the PM2.5 removal capacity of the air purification equipment should not be lower than the total infiltration PM2.5 load and indoor sources PM2.5 load (see Figure 11).

When using an air cleaner, the CADR of the air cleaner can be selected as follows:

(22) $$CADR\geq \frac{M}{C_{n}}$$

where *CADR* is the volume of clean air (m^3^/h).

At present, in addition to the coarse filter in China’s standards, filtration efficiency of the air filter generally adopts the counting efficiency, rather than the arrestance. In order to facilitate the selection of air filters, the research team measured the weight efficiency of different levels of air filters, as listed in Table 6, which can be used for equipment selection of indoor PM2.5 pollution control design. Designers can also use a series combination of air filters of different grades according to Table 6.

## 5. Control Measures and Energy-Saving Operation Strategy

### 5.1. Passive Control Measures

#### 5.1.1. Building Entrance and Exit

Some measures should be taken in building entrances and exits to block PM2.5 from entering the room. The most common measures include the installation of wind porch, door bucket, revolving door, telescopic automatic door, air curtain, door closers, improving the airtightness of the outer door, and using dust floor mats in the entrance of the building.

#### 5.1.2. External Window and Curtain Wall

The external window and curtain wall are the main ways to prevent the penetration of outdoor PM2.5. Due to the strict design and construction requirements of modern buildings, except for the necessary holes, there are almost no gaps or holes in the building walls, which enhances the airtightness of the external windows and curtain walls of the building, and can block the penetration of outdoor PM2.5 into the room. Among them, T/ASC 02-2016 Assessment Standard for Healthy Building stipulates that for areas with an air quality index below 100 per year in more than 310 days, the airtightness of the external window should reach specified level 4 in GB/T 7106-2008 Graduations and Test Methods of Air Permeability, Watertightness, Wind Load Resistance Performance for Building External Windows and Doors. The external window airtightness of other areas should reach level 6 or above. In addition, the curtain wall meets the specified level 3 and above in GB/T 21086-2007 Curtain Wall for Building [16,20,21].

The airtight performance of the external door and window will affect the penetration of the outdoor PM2.5 into the room. Although the external door and window with high airtight performance is adopted in design, if the product quality or construction quality is not high, the airtightness of the external door and window will not meet the design requirements. Thus, it is necessary to carry out key inspections on the sealing parts of the external doors and windows.

In addition, when the outdoor air quality is excellent, it is preferable to use natural ventilation to purify and improve the quality of the indoor air.

#### 5.1.3. External Walls

The gaps and holes in external walls will affect the airtightness of the entire building, which are filled and sealed. Some common gaps include the following: the gap between a split air conditioner or a range hood and the outdoor connected pipe hole, the chimney hole for a household gas wall-hung boiler wall or a window, the gap between the frame and the window or door, etc.

### 5.2. Active Control Measures

#### 5.2.1. Equipment with a Large Amount of PM2.5 Emission

Printers, copiers, and other equipment with a large amount of PM2.5 emissions will generate PM2.5 and other contaminants during operation. These devices should be kept at a certain distance from humans, for example, in a dedicated room. In order to reduce the exposure of PM2.5 for the personnel using the equipment and to prevent PM2.5 from collapsing into other spaces, it is advisable to take some preventative measures such as having an exhaust air flow in the room or area where a large number of PM2.5 equipment is distributed.

#### 5.2.2. Smoking, Burning of Mosquito Coil, Kitchen Fumes

Indoor smoking, burning of a mosquito coil, and kitchen fumes, etc., can increase the PM2.5 concentration in a short period. Without effective control measures and under normal outdoor air volume conditions, the air purification equipment cannot purify the PM2.5 load immediately. Therefore, in order to ensure that indoor PM2.5 pollution can be controlled, some effective measures should be done, such as prohibiting indoor smoking, setting up smoking isolation areas, prohibiting the use of burning mosquito coils, and effective soot emissions design for the kitchen, etc.

#### 5.2.3. Ventilation Air Purification System

A mechanical ventilation air purification system is used when natural ventilation does not meet the requirements. In this case, the outdoor air intake should be away from outdoor PM2.5 pollution sources, i.e., outdoor smoking areas, chimneys, exhaust vents and exhaust hoods (especially kitchen exhaust), and building materials processing rooms (tiles, stone, and wood, etc.). Moreover, installation of an air purifier for the ventilation system or setting up a separate air cleaner in the room can be beneficial.

### 5.3. Energy-Saving Operation Strategy

Outdoor PM2.5 pollution has the characteristic of randomness. When the outdoor air quality is good and the air purification equipment is still in a working state, the energy consumption of the fan and the operating cost is increased. Therefore, the energy-saving operation strategy of a PM2.5 pollution control system should be formulated according to the characteristics of a PM2.5 pollution control system and the variation characteristic of indoor and outdoor PM2.5 pollution. For example, installing a PM2.5 test instrument indoors or installing a monitoring device linked to a purification equipment can be effective solutions.

**Figure 12:**
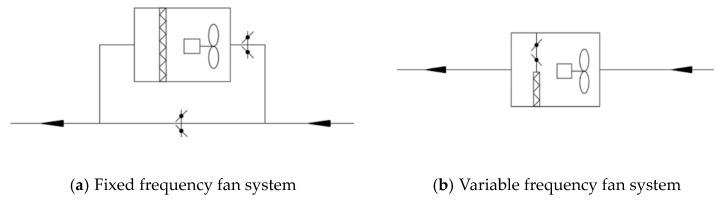
PM2.5 pollution control system bypass solution.

**Figure 13:**
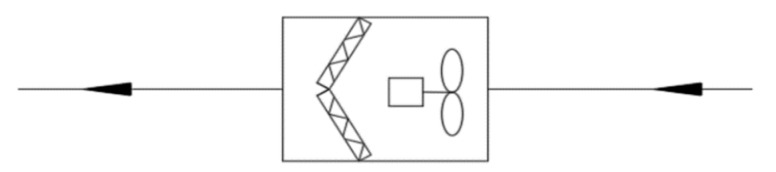
PM2.5 pollution control system opening and closing plan.

For centralized and semi-centralized air conditioning systems, according to the outdoor PM2.5 pollution, the air filter can be operated in the following two ways: (1) When the outdoor PM2.5 concentration is higher than the indoor calculated concentration, the filter is put into operation and (2) when the outdoor PM2.5 concentration is lower than the indoor calculated concentration, the filter is not put into operation. Users could open, bypass, or close the air filter according to the outdoor PM2.5 pollution. At the same time, depending on filter operation, whether it is on or off, the fan has a corresponding automatic frequency conversion adjustment function to achieve an energy-saving operation (see Figure 12 and Figure 13) [14].

## 6. Conclusions and Recommendations for Future Work

PM2.5 is regarded as an important pollutant affecting the quality of the air environment. Indoor and outdoor PM2.5 pollution control is an important project, which still needs various related institutions (governments, scientific research institutions, design units, universities, real estate developers, related product manufacturers, medical service industries, etc.) to work together to reduce the indoor and outdoor PM2.5 pollution levels, improve the quality of the building living environment, and therefore people’s health and happiness index. From this paper, the following conclusions and recommendations for further work are presented:

1. We propose the outdoor calculated concentration of PM2.5 in 74 cities in China using “not guaranteed for five days” method, which is based on the outdoor PM2.5 concentration monitoring data from 2016 to 2018. In addition, different levels of indoor calculated concentrations of PM2.5 (25 μg/m^3^, 35 μg/m^3^, are 75 μg/m^3^) are proposed.
2. Methods for selecting air purification equipment in centralized, semi-centralized and decentralized air conditioning systems are proposed. The air purification equipment in the OAHU gives priority to setting up centrally and purifying all indoor PM2.5 loads.
3. PM2.5 pollution control measures and system energy-saving operation strategies in buildings are put forward, such as passive control measures (building entrance and exit, external window and curtain wall, and external wall), active control measures (air filter and air cleaner), and energy-saving operation strategy (air filter bypass), etc.
4. We provide a valuable reference for engineering design, construction, and operation of a PM2.5 pollution control system. Future works could focus on experimental verification, effective indoor PM2.5 pollution control design and evaluation methods, innovative control technologies and products, operation and maintenance, and management strategies, etc.
5. From the perspective of a long-term developmental strategy, there is still much work to be done to combat air pollution. For example, we should take a series of measures to strengthen the comprehensive control of air pollution in industrial enterprises, promote the control of bulk coal and the reduction and replacement of coal consumption, carry out special rectification of the emissions of diesel trucks exceeding the national standards, strengthen land greening and dust control, and develop more efficient energy systems, etc.

## Figures and Tables

**Table 1:** Seventy-four cities’ outdoor calculated concentrations of PM2.5 in China.

City	Outdoor Calculated Concentration of PM2.5 (μg/m^3^)	City	Outdoor Calculated Concentration of PM2.5 (μg/m^3^)	City	Outdoor Calculated Concentration of PM2.5 (μg/m^3^)	City	Outdoor Calculated Concentration of PM2.5 (μg/m^3^)	City	Outdoor Calculated Concentration of PM2.5 (μg/m^3^)
Haikou	54	Nanning	93	Jiaxing	121	Shenyang	149	Harbin	206
Lhasa	58	Guangzhou	96	Chengde	123	Qingdao	149	Tianjin	212
Xiamen	62	Zhangjiakou	98	Nantong	125	Zhenjiang	149	Taiyuan	229
Kunming	64	Zhaoqing	99	Hangzhou	126	Taizhou	150	Tangshan	229
Shenzhen	65	Jiangmen	100	Huzhou	127	Wuhan	151	Langfang	240
Fuzhou	66	Xining	101	Shaoxing	128	Huai’an	151	Beijing	241
Huizhou	68	Foshan	103	Wuxi	130	Hefei	152	Hengshui	253
Zhoushan	71	Ningbo	106	Yinchuan	131	Changzhou	154	Xi’an	261
Zhuhai	71	Quzhou	106	Changchun	132	Qinghuangdao	154	Baoding	267
Wenzhou	82	Jinhua	113	Chongqing	133	Changsha	156	Urumqi	276
Guiyang	84	Shanghai	113	Nanjing	134	Chengdu	165	Zhengzhou	277
Taizhou	84	Nanchang	115	Suzhou	134	Suqian	170	Handan	283
Zhongshan	85	Hohhot	118	Lianyungang	144	Xuzhou	195	Xingtai	288
Lishui	87	Dalian	120	Yangzhou	147	Ji’nan	203	Shijiazhuang	344
Dongguan	92	Lanzhou	120	Yancheng	148	Cangzhou	206		

Note: The original statistical datas are derived from the China Air Quality Online Monitoring and Analysis Platform. The statistical interval is 2016~2018.

**Table 2:** Indoor calculated concentration of PM2.5, in China.

Class	Current Value (μg/m^3^)	Guiding Value (μg/m^3^)
I	35	25
II	75	35

Note: Indoor calculated concentration of PM2.5 is the daily average value.

**Table 3:** PM2.5 external window penetration coefficient *P.*

Airtightness Level	4	5	6	7	8
PM2.5 external window penetration coefficient *P*	0.85	0.80	0.75	0.70	0.70

**Table 4:** Air permeability performance classification of external window.

Airtightness Level	4	5	6	7	8
*Q*_1_/(m^3^/(m·h))	2.5 ≥ *Q*_1_ > 2.0	2.0 ≥ *Q*_1_ > 1.5	1.5 ≥ *Q*_1_ > 1.0	1.0 ≥ *Q*_1_ > 0.5	*Q*_1_ ≤ 0.5
*Q*_2_/(m^3^/(m^2^·h)	7.5 ≥ *Q*_2_ > 6.0	6.0 ≥ *Q*_2_ > 4.5	4.5 ≥ *Q*_2_ > 3.0	3.0 ≥ *Q*_2_ > 1.5	*Q*_2_ ≤ 1.5

**Table 5:** Air change rates corresponding to different airtight external windows [17,18].

Airtightness Level	4	5	6	7	8
Air change rate (h^−1^)	0.60~0.80	0.55~0.65	0.40~0.55	0.25~0.40	0.10~0.25

**Table 6:** PM2.5 arrestance of air filter [13,14,19].

Air Filter Class (European Standard/Chinese National Standard)	Arrestance (%)	Air Filter Class (European Standard/Chinese National Standard)	Arrestance (%)
G3/C1	1.29	F7/GZ	47.22
G4/Z3	20.53	F8/GZ	59.29
M5/Z2	27.26	F9/GZ	84.10
M6/Z1	45.22	H10/YG	91.82
